# Long non-coding RNA KCNQ1OT1 facilitates the progression of cervical cancer and tumor growth through modulating miR-296-5p/HYOU1 axis

**DOI:** 10.1080/21655979.2021.1982230

**Published:** 2021-10-27

**Authors:** Jun Liu, Yingmei Wang

**Affiliations:** a Department of Obstetrics and Gynecology, Tianjin Medical University General Hospital, Tianjin, China; b Department of Obstetrics and Gynecology,Hohhot First Hospital, Hohhot, Inner Mongolia, China

**Keywords:** LncRNA kcnq1ot1, miR-296-5p, hyou1, wnt/β-catenin, cervical cancer

## Abstract

Literature reports that lncRNA KCNQ1OT1 is markedly up-regulated in cervical cancer (CC) tissues and cell lines, and KCNQ1OT1 can promote the proliferation and metastasis of CC cells. This current work was designed to investigate the molecular mechanism underlying the participation of KCNQ1OT1 in CC progression. Herein, RT-qPCR was utilized for determining the levels of KCNQ1OT1, miR-296-5p and HYOU1 in clinical tumor tissue specimens and CC cell lines. Then, starBase predicted the complementary binding sites of KCNQ1OT1 and miR-296-5p or miR-296-5p and HYOU1. Dual-luciferase reporter assay/RIP assay validated the interplays among KCNQ1OT1/miR-296-5p/HYOU1. In addition, CCK-8, wound healing and transwell assays were employed to assess the proliferative, migrative and invasive properties of CC cells. Moreover, nude mice xenograft model was established by subcutaneously injection with SiHa cells in order to validate the precise functions of KCNQ1OT1/miR-296-5p/HYOU1 axis in CC *in vivo*. Besides, Immunohistochemical staining examined Ki-67 expression in xenograft tumors and western blotting analysis detected expressions of MMP2/9 and Wnt/β-catenin signaling pathway in CC cells and xenograft tumors. Elevated KCNQ1OT1 and HYOU1 as well as reduced miR-296-5p were observed in clinical tumor tissue specimens and CC cell lines. Results revealed that upregulation of miR-296-5p counteracted the enhancing effects of overexpressed KCNQ1OT1 on the proliferative, migrative and invasive abilities of CC cells. Additionally, HYOU1 overexpression abolished the suppressing effects of silenced KCNQ1OT1 on the malignant behaviors of CC cells and tumor growth. To conclude, KCNQ1OT1 could aggravate the malignant behaviors of CC and facilitate tumor growth through modulating miR-296-5p/HYOU1 axis.

## Introduction

Cervical cancer (CC), a highly prevalent malignancy, seriously endangers physical and mental health in women. In recent years, it has been widely concerned as a public health problem, and its high incidence and trends in younger generation also attract increasing attention [1]. Statistical data indicate that the global number of new cases of CC is approximately 570,000 in 2018 [2]. It is well known that surgery, radiotherapy and chemotherapy are conventional therapeutics for CC clinically [3]. The therapeutic outcomes of CC are closely associated with its histological type and lymph node metastasis [4]. In order to improve the early diagnosis rate of CC and ensure effective prognosis of patients, the scholars devote themselves to explore novel tumor markers with higher specificity [5,6].

Long non-coding RNA (lncRNA) belongs to non-coding RNA family comprising nucleotide (nt) sequences longer than 200 nts [7]. Through regulating the expression of genes at chromatin modification, transcriptional and post-transcriptional levels, it can greatly influence tumor development [8]. It has been reported that lncRNA KCNQ1 overlapping transcript 1 (KCNQ1OT1) is abnormally expressed in multiple cancers and closely linked to tumor progression [9,10]. Importantly, KCNQ1OT1 has been proved to be markedly up-regulated in CC tissues and cell lines, and KCNQ1OT1 overexpression can promote the proliferation and metastasis of CC cells [11]. Hence, this study further investigated the molecular mechanism underlying the participation of KCNQ1OT1 in the development of CC.

Studies indicate that a variety of lncRNAs have a sponge adsorption effect on miRNAs and thus participate in the process of tumor development [12,13]. miRNAs are approximately 22 nt non coding RNAs, which regulate gene expression in a sequence-specific manner via translational inhibition or mRNA degradation [14]. Li et al. [15] proved that KCNQ1OT1 was an upstream regulator of miR-296-5p in neuroblastoma cells. As a highly conserved miRNA, miR-296-5p has been shown to be down-regulated in CC patients. Besides, miR-296-5p possibly inhibited the oncogenic influences of high mobility group A in CC [16]. Meanwhile, Xie et al. [17] verified that the expression of miR-296-5p was decreased in CC cell lines, and up-regulation of miR-296-5p suppressed the proliferation, invasion, migration and EMT of CC cell lines.

Then, we predicted that the 3ʹ-UTR of Hypoxia upregulated protein 1 (HYOU1) was complementary to the seed sequence of miR-296-5p. HYOU1 has been identified as a gene specifically expressed under multiple malignant conditions in man [18]. Zhou et al. [19] reported that HYOU1 was highly expressed in tissues of NPC and linked closely to decreased progression-free survival (PFS) and overall survival (OS). Additionally, HYOU1 is also highly associated with expansion and metastatic activities of epithelial ovarian tumor cell lines [18].

Wnt/β-catenin pathway is ubiquitous within organisms, acting as a critical signaling pathway regulating cell proliferation and differentiation [20]. Wnt/β-catenin signaling pathway is tightly linked to the occurrence and evaluation of CC. Moreover, abnormal activation of this pathway leads to the occurrence, development and metastasis of CC [21]. Recent studies have reported that lncRNA KCNQ1OT1 can affect tumor development and progression via modulation of gene expression in Wnt/β-catenin signaling pathway [22,23].

Herein, the levels of lncRNA KCNQ1OT1, miR-296-5p and HYOU1 in clinical tumor tissue specimens and CC cell lines were assessed and the influences of lncRNA KCNQ1OT1/miR-296-5p/HYOU1 on CC cell proliferation, migration, invasion as well as tumor growth were evaluated, so as to determine the specific role of lncRNA KCNQ1OT1 and explore the molecular mechanism underlying the participation of KCNQ1OT1 in the development of CC. Additionally, the biological function of Wnt/β-catenin signaling pathway was also investigated in this work.

## Materials and methods

### Tissue sample collection/handling

Patients diagnosed with CC at Hohhot First Hospital from 2018 to 2019 were chosen for this investigation. CC tumor tissue specimens and adjacent normal tissues were extracted from 10 patients. All study participants were informed in advance and submitted written consents. The present study was authorized by the Ethics Committee of Hohhot First Hospital.

### Cell culture

Cervical cancer-derived cell lines HeLa (HPV18), SiHa (HPV16), C33A (HPV-negative) and normal human cervical epithelial cell line End1/E6E7 were purchased from the Cell Bank of the Chinese Academy of Science (Shanghai, China). Cells were cultured in Dulbecco’s modified Eagle’s medium containing 10% FBS (Gibco, NY, USA), 100 U/ml penicillin and 0.1 mg/ml streptomycin (Sigma-Aldrich, MO, USA) at 37°C under a 5% CO_2_ atmosphere.

### Cell transfection

The pcDNA-KCNQ1OT1, pcDNA-NC, miR-296-5p mimic, miR-NC, sh-KCNQ1OT1, sh-NC, HYOU1 overexpression vector and the control vector were bought from GenePharma Corporation (Shanghai, China). When the cells reached 70% confluence, shRNA or plasmid was transfected using Lipofectamine 2000 (Invitrogen, CA, USA) under manufacturer’s guidance. In brief, the vectors and Lipofectamine 2000 were separately diluted with Opti-MEM (Invitrogen, CA, USA) and incubated for 5 min at room temperature. The two mixtures were mixed and incubated for 20 min at room temperature. Then, the transfection mixture was added to each well and incubated for 6 h in serum-free medium. Next, the serum-free medium was replaced by the complete medium and cultured for 48 h prior to further experiments.

### Nude mice Xenograft model

The special pathogen-free grade (SPF) BALB/c nude mice (4–6 weeks) were procured from Animal Center of Chinese Academy of Medical Sciences (Beijing, China). Briefly, SiHa cells received designed transfection, and then the transfected SiHa cells were suspended in PBS at a density of 1 × 10^7^/ml. Cell suspensions (200 μl) were injected subcutaneously into nude mice. The tumor sizes were measured at 3-day intervals, and mice were executed on day 30 post the injection of SiHa cells. The tumor tissues were excised for subsequent analysis. All animal experiments were approved by the Institutional Animal Care and Use Committee of Hohhot First Hospital. This research followed the Guidelines for the Care and Use of Laboratory Animals and ‘3 R’ principle.

### Cell counting kit-8 (CCK-8) assay

Viability of SiHa cells was evaluated by performing CCK-8 assay. Cells (5 × 10^3^ cells/well) were seeded on 96-well plates. 10 µl CCK-8 reagent (Beyotime, Shanghai, China) was added into each well and incubated for 2 h at 37°C. Finally, detection of optical density (OD) at 450 nm was carried out with a microplate-reader (Bio-Rad, CA, USA).

### Wound healing assay

The migration ability of SiHa cells was evaluated by performing wound healing assay. In short, cells were seeded on 6-well plates and grown to 90% confluence. Afterward, the cell monolayers were scratched with 200-μl pipette tips for obtaining horizontal wounds. Then, cells were washed twice with PBS and cultured in fresh serum-free DMEM. Finally, an optical microscope (Leica, Wetzlar, Germany) was applied to observe the wounds at 0 and 24 h.

### Transwell invasion assay

The invasive ability of SiHa cells was evaluated by performing transwell invasion assay. Cells were suspended into fresh serum-free DMEM. Subsequently, 1 × 10^5^ cells were seeded into the upper chambers of transwell plates pre-coated with matrigel (BD Biosciences, NJ, USA). The medium containing 10% FBS was added into the lower chambers. Post 48 h of incubation, noninvasive cells were removed using cotton swabs. Then, cells on the lower surface were fixed in 4% paraformaldehyde and subsequently stained with 0.1% crystal violet (Solarbio, Beijing, China). Finally, an optical microscope (Leica, Wetzlar, Germany) was used for photographing the invaded cells.

### Reverse transcription-quantitative PCR (RT-qPCR)

RNAiso Plus reagent (TaKaRa, Tokyo, Japan) was employed for extracting total RNA under manufacturer’s guidance. The purity and concentration of RNA were evaluated by an ultraviolet spectrophotometer (Bio-Rad, CA, USA). Afterward, total RNA was reversely transcribed into cDNA with PrimeScript RT reagent kit (TaKaRa, Tokyo, Japan). Next, quantitative analysis was performed for determining mRNA expression on an ABI 7500 system (Applied Biosystems, CA, USA) by SYBR Premix Ex Taq kit (TaKaRa, Tokyo, Japan). GAPDH served as the endogenous control for KCNQ1OT1 and HYOU1 and U6 served as the endogenous control for miR-296-5p. The sequences of the primers were as follows: KCNQ1OT1: forward, 5′- GCACTCTGGGTCCTGTTCTC −3′ and reverse, 5′- CACTTCCCTGCCTCCTACAC −3′; miR-296-5p: forward, 5′- CGTCTATACAGACCCTGGCTTTTC −3′ and reverse, 5′- CTCAACTGGTGTCGTGGA-3′; HYOU1 5ʹ-CTTCCACATCAACTACGGCG-3ʹ, reverse 5ʹ-CTCTTCTGCGCTGTCCTCTA-3ʹ; GAPDH: forward, 5′- GAAGGTGAAGGTCGGAGTC −3′ and reverse, 5′- GAAGATGGTGATGGGATTTC −3′; U6: forward, 5ʹ- CGAGCACAGAATCGCTTCA −3ʹ and reverse, 5ʹ- CTCGCTTCGGCAGCACATAT −3ʹ. The PCR conditions were as follows: 95°C for 10 min, followed by 42 cycles of 95°C for 15 sec and 58°C for 60 sec. Finally, relative expression levels were calculated using 2^–ΔΔCT^ methods [24].

### Western blotting analysis

RIPA lysis buffer (Beyotime, Shanghai, China) was applied for extraction of proteins from tissues and cells. First of all, 10% sodium dodecyl sulfate (SDS)-polyacrylamide gel electrophoresis was adopted to segregate lysed proteins. Then, separated proteins were transferred onto PVDF membranes (Millipore, MA, USA). Membranes were blocked in 5% skimmed-milk at room temperature for 1 h. Afterward, membranes were incubated with primary antibodies at 4°C overnight: anti-MMP2 (Abcam, ab181286, 1:1000), anti-MMP9 (Abcam, ab228402, 1:1000), anti-β-catenin (Abcam, ab32572, 1:5000), anti-c-myc (Abcam, ab32072, 1:1000), anti-cyclin D1 (Abcam, ab134175, 1:10,000), anti-HYOU1 (Abcam, ab134944, 1:1000), anti-GAPDH (Abcam, ab181602, 1:10,000). On the second day, membranes were incubated with appropriate horseradish peroxidase-conjugated secondary antibodies (Abcam, ab205718, 1:50,000) at room temperature for 1 h. Finally, signals of immunoblots were developed an enhanced chemiluminescence (ECL) kit (Millipore, MA, USA).

### Immunohistochemical staining

The tumor tissues were fixed in 4% paraformaldehyde at room temperature for 24 h, embedded in paraffin and then sliced into 4-µm slices. Next, these slices were subjected to drying, deparaffinization and rehydration. The slices were restored with sodium citrate and incubated with 3% H_2_O_2_ for 10 min. Afterward, the slices were blocked in BSA and then subjected to incubation with primary antibody including Ki-67 (Abcam, ab21700, 1:200) overnight at 4°C. On the second day, secondary antibody (Abcam, ab205718, 1:2000) was employed to stain sections at room temperature for 2 h. Subsequently, the sections were stained with DAB mixture (Solarbio, Beijing, China), followed by counterstained with hematoxylin. The tissue sections after staining were examined using Image Pros Plus 5.0 software (Silver Springs, MD, USA).

### Dual-luciferase reporter assay

The wild and mutant types of KCNQ1OT1 mRNA 3′-UTR fragment containing miR-296-5p binding sites were amplified and cloned into the pGL3 dual-luciferase reporter plasmid (Promega, WI, USA). SiHa cells were co-transfected with constructs and miR-296-5p mimic or miR-NC for 48 h. Dual Luciferase Reporter Assay Kit (Promega, WI, USA) was employed for detection of relative luciferase reporter activities. The firefly luciferase activities were normalized to those of Renilla luciferase. A similar strategy was employed to assess HYOU1/miR-296-5p interplays.

### Anti-Argonaute 2 (Ago2) RNA immunoprecipitation (RIP) assay

RIP assay was conducted by the Magna RIP RNA-binding protein immunoprecipitation kit (Millipore, MA, USA) according to the manufacturer’s instructions. In brief, cells were lysed using RIP lysis buffer. Then, the lysates were incubated with magnetic beads conjugated to anti-Ago2 or anti-IgG at 4°C overnight. The immunoprecipitated RNA was then extracted and purified. The levels of miR-296-5p and HYOU1 were determined by RT-qPCR.

### Statistical analysis

Experimental data were expressed as mean values ± standard deviation (SD). One-way analysis of variance (ANOVA) followed by Tukey’s post hoc test was employed for statistical analysis among multiple groups and two-way ANOVA followed by Tukey’s post hoc test was applied to analyze the data presented in Figure 3(c), Figure 7(b) and Figure 7(c). Statistical differences between two groups were determined using unpaired Student’s t-test. There were three levels of p < 0.05, p < 0.01 and p < 0.001, and p < 0.05 was considered to be statistically significant difference.

## Results

### LncRNA KCNQ1OT1 was distinctly elevated in CC

**Figure 1.**
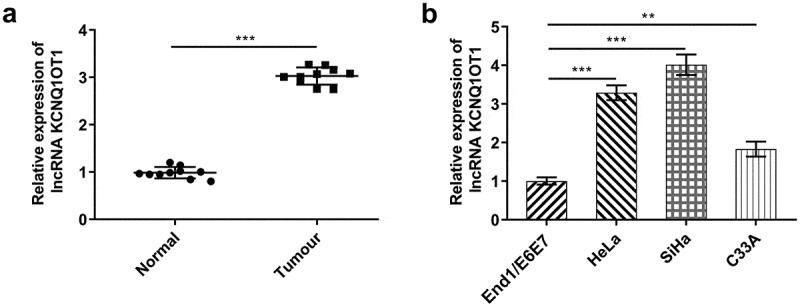
Elevated KCNQ1OT1 expression in CC. (a) RT-qPCR for determination of the expression differences of KCNQ1OT1 in CC tumor tissue specimens and adjacent non-tumor tissues. (b) RT-qPCR for determination of the expression differences of KCNQ1OT1 in CC cell lines (HeLa, SiHa, C33A) and normal human cervical epithelial cell line (End1/E6E7)

In order to crystallize the biological function of KCNQ1OT1 in CC, RT-qPCR was applied for evaluating the expression differences of KCNQ1OT1 in CC tumor tissue specimens and adjacent non-tumor tissues. Obviously, up-regulated KCNQ1OT1 expression was observed in tumor tissues in comparison with paracancerous tissues (Figure 1(a)). Besides, compared with that in normal human cervical epithelial cell line (End1/E6E7), KCNQ1OT1 expression was greatly up-regulated in CC cell lines (HeLa, SiHa, C33A) (Figure 1(b)). SiHa cells were chosen for subsequent studies in accordance with peak KCNQ1OT1 expression in SiHa.

### Down-regulation of KCNQ1OT1 suppressed CC cell proliferation, migration and invasion

**Figure 2.**
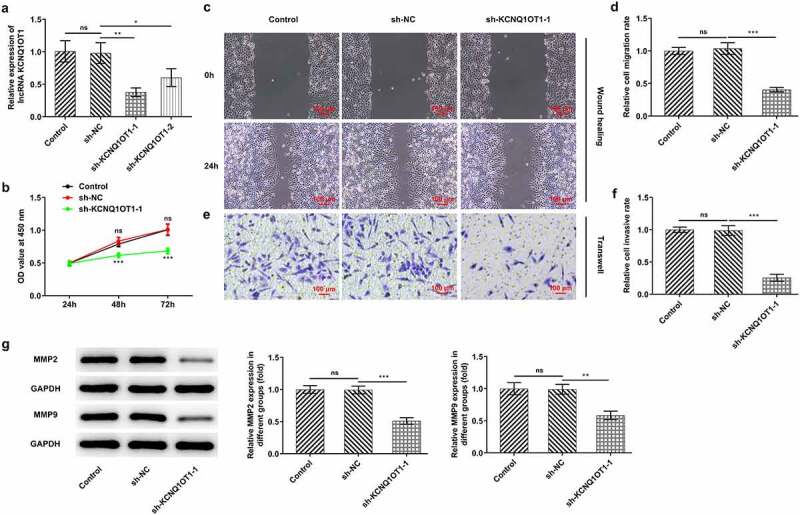
Silenced KCNQ1OT1 suppressed cell proliferation, migration and invasion in CC. (a) RT-qPCR validated interference efficacy in SiHa cells after transfection with KCNQ1OT1 knockdown lentiviral vectors. (b) CCK-8 assay examined the influence of KCNQ1OT1 silencing on CC cell proliferation. (c-d) Wound healing assay determined the influence of KCNQ1OT1 silencing on the migrative ability of CC cells. (e-f) Transwell assay detected the influence of KCNQ1OT1 silencing on the invasive ability of CC cells. (g) Western blotting analysis assessed the influence of KCNQ1OT1 silencing on the expressions of MMP2 and MMP9 in CC cells

In order to confirm the putative influences of KCNQ1OT1 on CC progression, KCNQ1OT1 knockdown lentiviral vectors were transfected into SiHa cells. RT-qPCR was employed for validating interference efficacy and sh-KCNQ1OT1-1 was selected for the further experiments due to a higher transfection efficacy (Figure 2(a)). Then, the proliferation, migration and invasion of SiHa cells were evaluated through application of CCK-8, wound healing as well as transwell assays. The results discovered that KCNQ1OT1 silencing exerted a remarkable inhibition on the proliferative ability of CC cells (Figure 2(b)). In addition, an obvious reduction in CC cell migrative (Figure 2(c, d)) and invasive rates (Figure 2(e, f)) was observed following KCNQ1OT1 knockdown. Moreover, the remarkably decreased expressions of MMP2 and MMP9 suggested that KCNQ1OT1 silencing led to suppressed cell migration and invasion in CC (Figure 2(g)).

### KCNQ1OT1 acted as a molecular sponge for miR-296-5p and negatively regulated miR-296-5p expression

**Figure 3.**
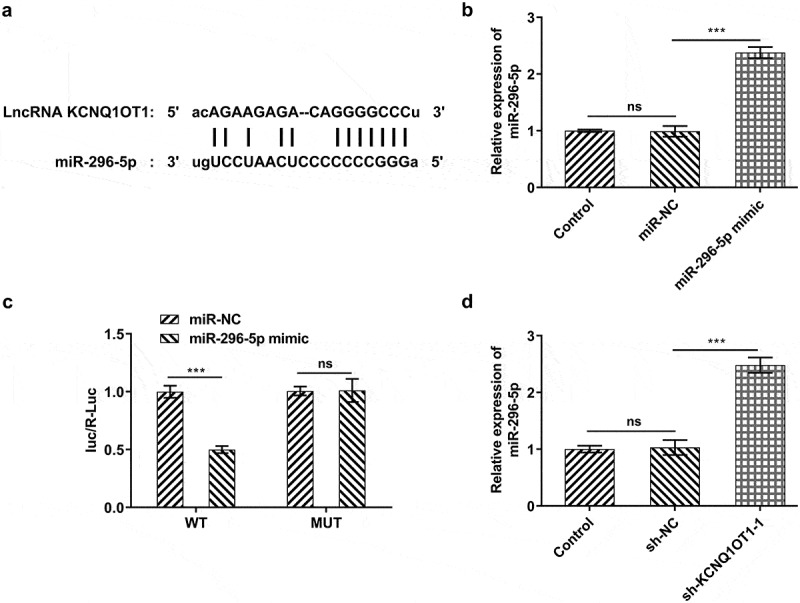
KCNQ1OT1 acted as a molecular sponge for miR-296-5p and negatively regulated miR-296-5p expression. (a) Bioinformatics analysis predicted the potential binding sites of miR-296-5p in KCNQ1OT1 sequence. (b) RT-qPCR validated overexpression efficacy in SiHa cells after transfection with miR-296-5p mimic. (c) Dual-luciferase reporter assay confirmed the binding relationship between KCNQ1OT1 and miR-296-5p. (d) RT-qPCR for determination of miR-296-5p level in SiHa cells after transfection with sh-KCNQ1OT1-1

LncRNAs are widely considered as ‘sponges’ of miRNAs. Bioinformatics analysis predicted the potential binding sites of miR-296-5p in KCNQ1OT1 sequence (Figure 3(a)). Then, miR-296-5p-mimic was transfected into SiHa cells and the transfection efficiency was verified by performing RT-qPCR (Figure 3(b)). miR-296-5p mimic inhibited luciferase activity of KCNQ1OT1-WT, whilst luciferase activity of KCNQ1OT1-MUT was unaffected following introduction of miR-296-5p mimic (Figure 3(c)). Furthermore, it was discovered that silenced KCNQ1OT1 effectively upregulated miR-296-5p expression in SiHa cells (Figure 3(d)).

### Decreased miR-296-5p expression in CC

**Figure 4.**
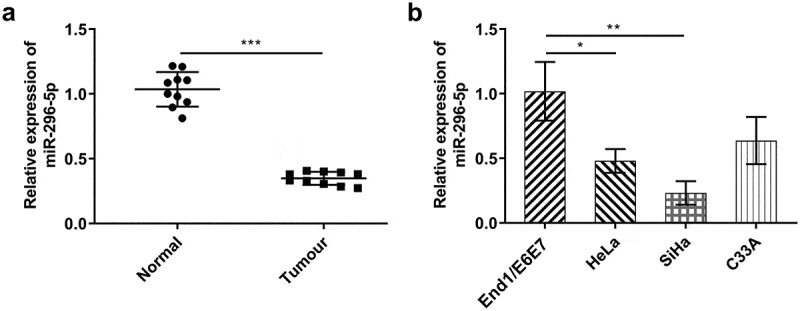
miR-296-5p was distinctly down-regulated in CC. (a) RT-qPCR for determination of the expression differences of miR-296-5p in CC tumor tissue specimens and adjacent non-tumor tissues. (b) RT-qPCR for determination of the expression differences of miR-296-5p in CC cell lines (HeLa, SiHa, C33A) and normal human cervical epithelial cell line (End1/E6E7)

In comparison with that in adjacent non-tumor tissues, miR-296-5p was notably down-regulated in CC tumor tissue specimens (Figure 4(a)). Meanwhile, miR-296-5p expression in CC cell lines (HeLa, SiHa, C33A) was distinctly decreased by comparison with human cervical epithelial cell line (End1/E6E7) (Figure 4(b)).

### Upregulation of miR-296-5p counteracted the enhancing effects of overexpressed KCNQ1OT1 on the proliferative, migrative and invasive abilities of CC cells

**Figure 5.**
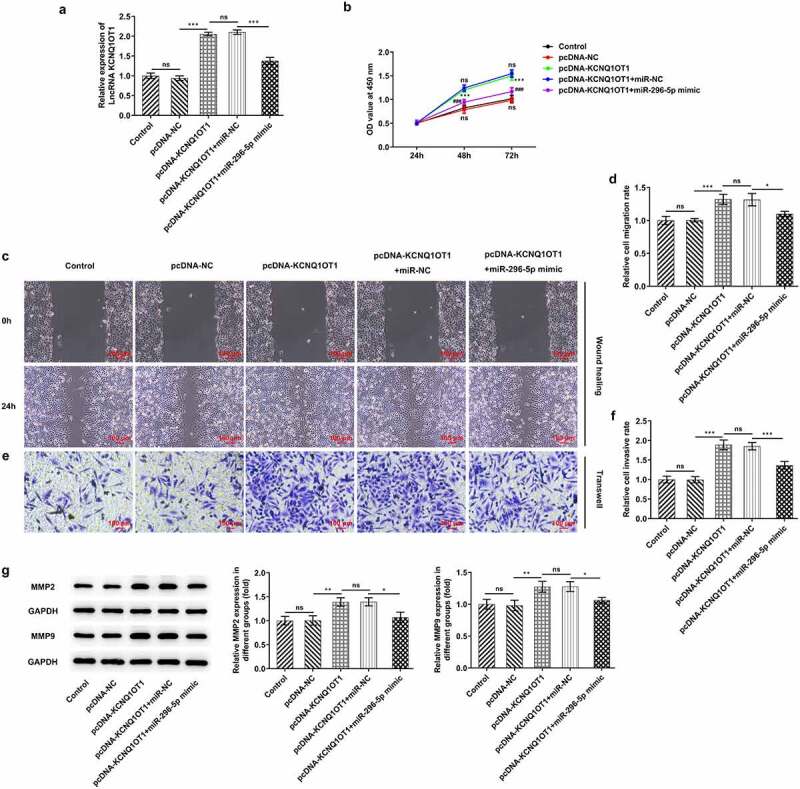
Upregulation of miR-296-5p counteracted the enhancing effects of overexpressed KCNQ1OT1 on the proliferative, migrative and invasive abilities of CC cells. (a) RT-qPCR for determination of KCNQ1OT1 level in SiHa cells after transfection with pcDNA-KCNQ1OT1 or co-transfection with miR-296-5p mimic. (b) CCK-8 assay examined the influence of miR-296-5p elevation on the enhanced proliferative ability of CC cells caused by KCNQ1OT1 overexpression. (c, d) Wound healing assay determined the influence of miR-296-5p elevation on the enhanced migrative ability of CC cells caused by KCNQ1OT1 overexpression. (e, f) Transwell assay detected the influence of miR-296-5p elevation on the enhanced invasive ability of CC cells caused by KCNQ1OT1 overexpression. (g) Western blotting analysis assessed the influence of miR-296-5p elevation on the increased expressions of MMP2 and MMP9 in CC cells caused by KCNQ1OT1 overexpression

To explore the biological function of miR-296-5p in the development of CC, SiHa cells were transfected with pcDNA-KCNQ1OT1 or co-transfected with miR-296-5p mimic. Transfection with pcDNA-KCNQ1OT1 greatly enhanced KCNQ1OT1 expression, which was partially abolished following co-transfection with miR-296-5p mimic (Figure 5(a)). KCNQ1OT1 overexpression boosted the proliferation of SiHa cells, which was reversed by miR-296-5p elevation (Figure 5(b)). Moreover, introduction of miR-296-5p mimic counteracted the enhancing effects of overexpressed KCNQ1OT1 on CC cell migration (Figure 5(c, d)) and invasion (Figure 5(e, f)). Furthermore, decreased expressions of MMP2 and MMP9 following co-transfection with miR-296-5p mimic further demonstrated that upregulation of miR-296-5p partly abolished the enhancing effects of KCNQ1OT1 overexpression on the migrative and invasive abilities of CC cells (Figure 5(g)).

### KCNQ1OT1 accounted for activating Wnt/β-catenin signaling pathway, which was enormously inactivated by overexpressed miR-296-5p

**Figure 6.**
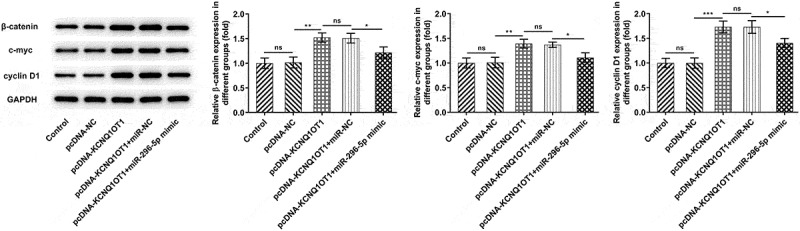
KCNQ1OT1 activated Wnt/β-catenin signaling pathway whereas miR-296-5p inactivated Wnt/β-catenin signaling pathway. Western blotting analysis examined the expressions of β-catenin, c-myc and cyclin D1 in SiHa cells after transfection with pcDNA-KCNQ1OT1 or co-transfection with miR-296-5p mimic

Next, Wnt/β-catenin signaling pathway was investigated, aiming to determine the regulating mechanism underlying KCNQ1OT1/miR-296-5p. Results revealed that overexpressed KCNQ1OT1 significantly increased the expressions of β-catenin, c-myc and cyclin D1 in SiHa cells, whereas β-catenin, c-myc and cyclin D1 were obviously down-regulated following co-transfection with miR-296-5p mimic (Figure 6).

### KCNQ1OT1 decoyed miR-296-5p to up-regulate its target gene HYOU1 in CC

**Figure 7.**
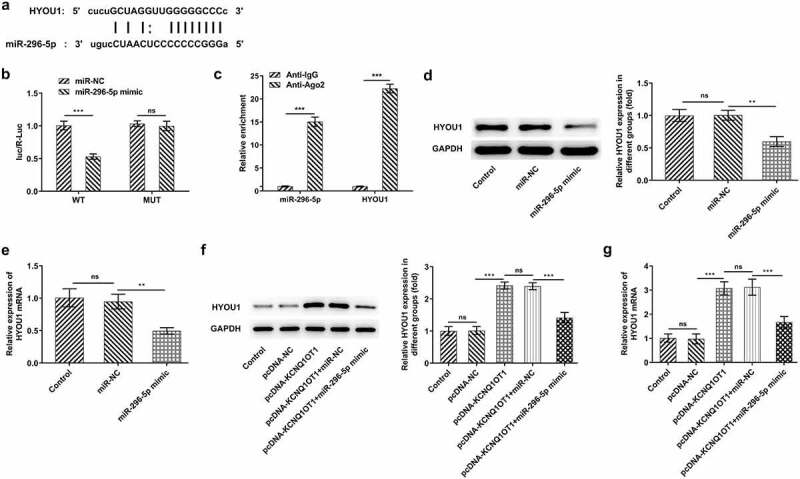
KCNQ1OT1 decoyed miR-296-5p to up-regulate its target gene HYOU1 in CC. (a) starBase predicted the complementary binding sites of miR-296-5p and HYOU1. (b) Dual-luciferase reporter assay confirmed the binding relationship between miR-296-5p and HYOU1. (c) RIP assay analyzed the potential binding between miR-296-5p and HYOU1. (d) Western blotting analysis examined the protein level of HYOU1 in CC cells after transfection with miR-296-5p mimic. (e) RT-qPCR detected the mRNA level of HYOU1 in CC cells after transfection with miR-296-5p mimic. (f) Western blotting analysis examined the protein level of HYOU1 in CC cells after transfection with pcDNA-KCNQ1OT1 or co-transfection with miR-296-5p mimic. (g) RT-qPCR detected the mRNA level of HYOU1 in CC cells after transfection with pcDNA-KCNQ1OT1 or co-transfection with miR-296-5p mimic

Afterward, starBase predicted the complementary binding sites of miR-296-5p and HYOU1, suggesting that HYOU1 may be a direct target of miR-296-5p (Figure 7(a)). Dual-luciferase reporter assay discovered that miR-296-5p mimic repressed luciferase activity of HYOU1-WT while it had no obvious influence on that of HYOU1-MUT, indicating the potential binding between miR-296-5p and HYOU1 (Figure 7(b)). Furthermore, Ago2-RIP assay was performed to evaluate the interactions between miR-296-5p and HYOU1 more directly. miR-296-5p and HYOU1 were abundantly detected in Anti-Ago2 group relative to Anti-IgG group (Figure 7(c)). It was observed that miR-296-5p overexpression down-regulated HYOU1 in CC cells (Figure 7(d, e)). Additionally, it was disclosed that the promoting effect of overexpressed KCNQ1OT1 on HYOU1 expression was abolished following co-transfection with miR-296-5p mimic (Figure 7(f, g)).

### HYOU1 overexpression visibly abolished the suppressing effects of silenced KCNQ1OT1 on CC cell proliferation, migration and invasion

**Figure 8.**
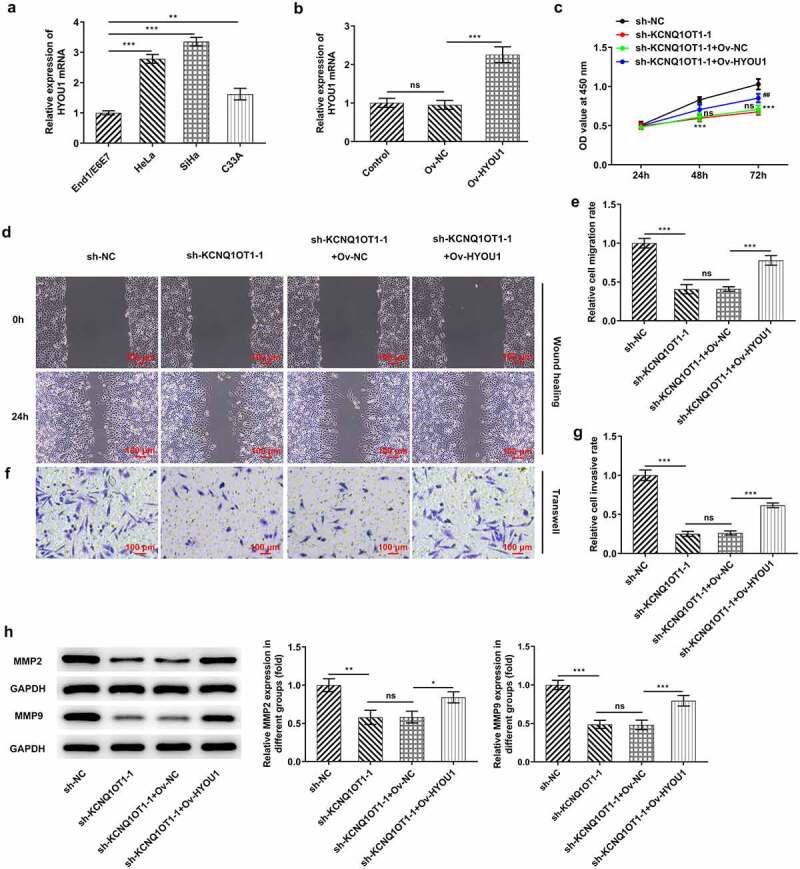
HYOU1 overexpression visibly abolished the suppressing effects of silenced KCNQ1OT1 on CC cell proliferation, migration and invasion. (a) RT-qPCR for determination of the expression differences of HYOU1 in CC cell lines (HeLa, SiHa, C33A) and normal human cervical epithelial cell line (End1/E6E7). (b) RT-qPCR verified the transfection efficiency in SiHa cells after transfection with Ov-HYOU1. (c) CCK-8 assay examined the influence of HYOU1 elevation on the suppressed proliferative ability of CC cells caused by silenced KCNQ1OT1. (d, e) Wound healing assay determined the influence of HYOU1 elevation on the suppressed migrative ability of CC cells caused by silenced KCNQ1OT1. (f, g) Transwell assay detected the influence of HYOU1 elevation on the suppressed invasive ability of CC cells caused by silenced KCNQ1OT1. (h) Western blotting analysis assessed the influence of HYOU1 elevation on the decreased expressions of MMP2 and MMP9 in CC cells caused by silenced KCNQ1OT1

Considering the modulating relationship among KCNQ1OT1, miR-296-5p and HYOU1, HYOU1 expression in CC cell lines (HeLa, SiHa, C33A) as well as human cervical epithelial cell line (End1/E6E7) was measured. In comparison with End1/E6E7 cells, HYOU1 was highly expressed in CC cell lines (Figure 8(a)). SiHa cells were transfected with Ov-HYOU1 and overexpression efficacy was verified by RT-qPCR (Figure 8(b)). Here, it was verified that the suppressing effect of KCNQ1OT1 knockdown on CC cell proliferation was reversed by HYOU1 overexpression (Figure 8(c)). Additionally, the obviously enhanced migrative and invasive abilities of CC cells upon HYOU1 elevation evidenced that overexpressed HYOU1 exacerbated CC cell migrative (Figure 8(d, e)) and invasive properties (Figure 8(f, g)), counteracting the suppressing effects of KCNQ1OT1 knockdown on CC cell migration and invasion. In addition, elevated MMP2 and MMP9 levels in SiHa cells after HYOU1 overexpression consistently confirmed this finding (Figure 8(h)).

### KCNQ1OT1 knockdown impeded tumor growth and repressed metastatic activity by downregulating HYOU1

**Figure 9.**
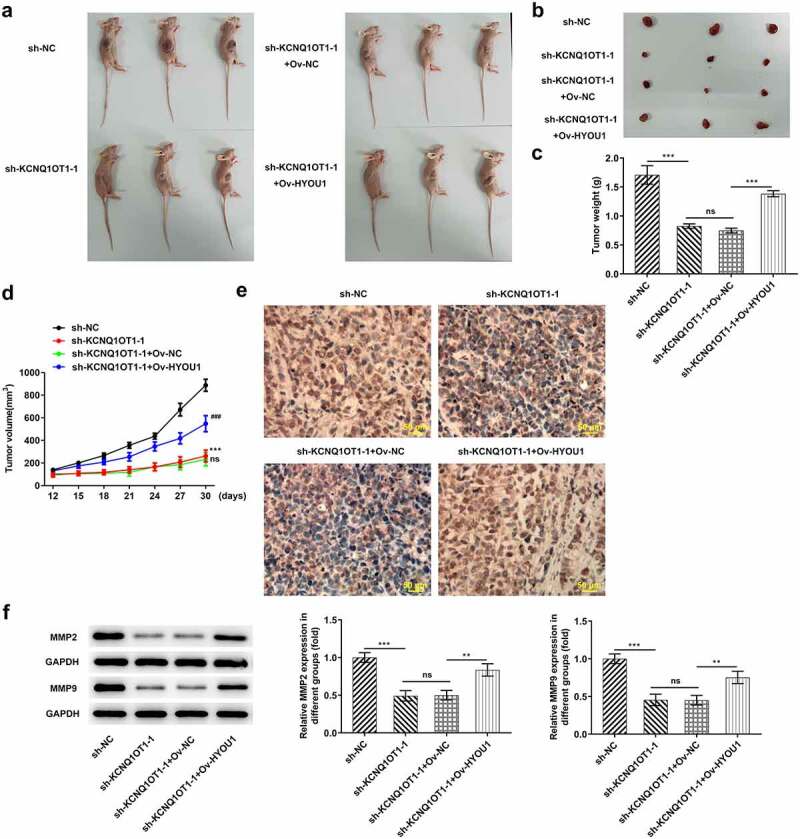
KCNQ1OT1 knockdown impeded tumor growth and metastasis by inhibiting HYOU1. (a) Representative morphologies of nude mice xenograft models. (b) Representative morphologies of xenograft tumors. (c) Weights of xenograft tumors. (d) Tumor volume curve. (e) Immunohistochemical staining examined Ki-67 expression in xenograft tumors. (f) Western blotting analysis detected expressions of MMP2 and MMP9 in xenograft tumors

In order to validate the precise functions of KCNQ1OT1 in CC, nude mice xenograft model was established by subcutaneously injection with SiHa cells transfected with sh-KCNQ1OT1-1 or co-transfected with Ov- HYOU1 (Figure 9(a)). Lower levels of tumor weight and tumor volume were observed in sh-KCNQ1OT1-1 group and this situation was counteracted following up-regulation of HYOU1 (Figure 9(b-d)). Moreover, Ki-67 expression in xenograft tumors was analyzed through Immunohistochemical staining and expressions of MMP2 and MMP9 in xenograft tumors were assessed by western blotting analysis. Elevated levels of Ki-67, MMP2 and MMP9 upon up-regulation of HYOU1 further evidenced that the inhibition of silenced KCNQ1OT1 on tumor growth and metastasis was partially abolished by HYOU1 elevation (Figure 9(e, f)).

### Overexpressed HYOU1 notably rescued the inactivating effect of KCNQ1OT1 knockdown on Wnt/β-catenin signaling pathway

**Figure 10.**
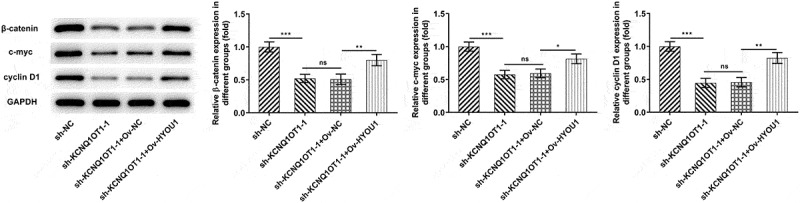
KCNQ1OT1 knockdown inactivated Wnt/β-catenin signaling pathway, which was abolished by HYOU1 overexpression. Western blotting analysis examined the expressions of β-catenin, c-myc and cyclin D1 in xenograft tumors

Wnt/β-catenin signaling pathway was further examined for assessing the molecular mechanism underlying KCNQ1OT1/miR-296-5p/HYOU1 axis in CC progression. Silenced KCNQ1OT1 remarkably reduced the expressions of β-catenin, c-myc and cyclin D1 in xenograft tumors, whereas β-catenin, c-myc and cyclin D1 were obviously enhanced upon HYOU1 elevation. HYOU1 overexpression activated Wnt/β-catenin signaling pathway in CC, abolishing the inhibition of KCNQ1OT1 silencing on Wnt/β-catenin signaling pathway (Figure 10).

## Discussion

Recently, CC is increasingly becoming a highly prevalent malignancy with high mortality, greatly threatening women’s health [1,2]. Due to the complex pathogenesis of CC, little progress has been made in CC therapies [3]. Researchers devote themselves to develop more precise therapeutics or targeted drugs to improve the curative effects for CC patients [25,26].

Evidence confirms that lncRNAs can be specifically expressed in tumor diseases or tissues. This feature is often used as a breakthrough point in tumor prediction, diagnosis, and target therapy in clinical or scientific research. KCNQ1OT1 has been proved to play a key role in the migration, invasion, metastasis and recurrence of cancers [9,10]. Duan et al. [27] reported that KCNQ1OT1 knockdown could inhibit the proliferative and invasive activities of colorectal cancer cells as well as enhance cell apoptotic ability via PI3K/AKT axis. Li et al. [28] confirmed the biological function of KCNQ1OT1 in bladder carcinoma (BC). Greatly elevated KCNQ1OT1 was observed in BC and KCNQ1OT1 can aggravate the malignant phenotypes of BC as well as accelerate the development of BC. In a word, KCNQ1OT1 may function to be an oncogene. Importantly, literature has verified that KCNQ1OT1 is highly expressed in CC patient cancerous tissues and CC cell lines and tightly associated with tumor volume increase, poor differentiation and radioresistance [11]. Consistent with previous studies, the present work also confirmed that KCNQ1OT1 was up-regulated in CC. Additionally, it was verified that silenced KCNQ1OT1 repressed the malignant behaviors of CC *in vitro* and *in vivo*.

A variety of lncRNAs have sponge adsorption effects on miRNAs [29]. Document described that KCNQ1OT1 acted as a molecular sponge for miR-296-5p and repressed miR-296-5p expression [15]. In this current work, starBase predicted the potential binding sites of miR-296-5p in KCNQ1OT1 sequence and dual-luciferase reporter assay confirmed the interplays between KCNQ1OT1 and miR-296-5p. Moreover, a distinct reduction of miR-296-5p expression was observed in CC patients [16]. Accumulating evidence demonstrated that loss of miR-296-5p boosted the occurrence of CC, accelerated the progression of clinical stage and increased the risk of recurrence [16,30]. Here in our research, miR-296-5p was decreased in cervical tumor tissues and CC cell lines, which was in conformity with abovementioned studies. In addition, the enhancement of KCNQ1OT1 overexpression on CC cell proliferation, migration and invasion were counteracted upon miR-296-5p elevation.

HYOU1 belongs to the heat shock protein 70 family that is widely expressed in many different cell types [31]. HYOU1 has been found to be remarkably up-regulated in some human cancers such as ovarian cancers and breast cancers [18,32]. Then, starBase predicted the binding site between miR-296-5p and HYOU1. Dual-luciferase reporter assay and Ago2-RIP assay proved the interactions between miR-296-5p and HYOU1. Up-regulation of KCNQ1OT1 enhanced HYOU1 expression, which was abolished by overexpressed miR-296-5p. Moreover, HYOU1 was up-regulated in CC cell lines. HYOU1 overexpression counteracted the inhibition of KCNQ1OT1 knockdown on the malignant behaviors of CC and tumor growth. To sum up, KCNQ1OT1 could accelerate CC progress through sponging miR-296-5p to elevate HYOU1.

In recent years, accompanied by in-depth research on the pathogenesis of CC, studies on cellular signaling pathways have received increasing attention [21]. Abnormal activation of Wnt signaling pathway can boost tumorigenesis and tumor progression through activating target genes like c-myc, cyclin D1, etc. [33]. Understanding of the interactions among lncRNAs, miRNAs and Wnt/β-catenin signaling pathway in CC may contribute to elucidation of molecular mechanisms for personalized therapy of CC [34,35]. Most notably, KCNQ1OT1 could facilitate proliferation/migration of ovarian cancer cells by up-regulating β-catenin [22]. Sun et al [36] reported that miR-296-5p functioned as a tumor suppressor via modulating Wnt/β-catenin signaling pathway in breast cancer. Consistent with abovementioned findings, results in our research clearly stated that KCNQ1OT1 activated Wnt/β-catenin signaling pathway by targeting miR-296-5p to enhance HYOU1 expression in CC.

## Conclusion

To sum up, this current work validated the biological roles and interplays of KCNQ1OT1/miR-296-5p/HYOU1 in CC progression. Herein, results revealed that KCNQ1OT1 could aggravate the malignant behaviors of CC and facilitate tumor growth through modulating miR-296-5p/HYOU1 axis, consequently developing promising molecular targets for CC therapeutics. In spite of the above achievement, in-depth research referring to clinical analysis should be further conducted to support these findings and excavate the predictive values of KCNQ1OT1/miR-296-5p/HYOU1.

## Data Availability

The analyzed data sets during the present study are available from the corresponding author on reasonable request.
